# The Kronos Early Estrogen Prevention Study (KEEPS): what have we learned?

**DOI:** 10.1097/GME.0000000000001326

**Published:** 2019-04-01

**Authors:** Virginia M. Miller, Fredrick Naftolin, Sanjay Asthana, Dennis M. Black, Eliot A. Brinton, Matthew J. Budoff, Marcelle I. Cedars, N. Maritza Dowling, Carey E. Gleason, Howard N. Hodis, Muthuvel Jayachandran, Kejal Kantarci, Rogerio A. Lobo, JoAnn E. Manson, Lubna Pal, Nanette F. Santoro, Hugh S. Taylor, S. Mitchell Harman

**Affiliations:** 1 Departments of Surgery and Physiology and Biomedical Engineering, Mayo Clinic, Rochester, MN; 2 Department of Obstetrics and Gynecology, New York University School of Medicine, New York, NY; 3 Education and Clinical Center, William S. Middleton Memorial Veterans Hospital, University of Wisconsin School of Medicine and Public Health and the Geriatric Research, Madison, WI; 4 Department of Epidemiology and Biostatistics, School of Medicine, University of California, San Francisco, CA; 5 Utah Lipid Center, Salt Lake City, UT; 6 Department of Medicine, Los Angeles Biomedical Research Institute at Harbor-University of California Los Angeles, Torrance, CA; 7 Department of Obstetrics, Gynecology and Reproductive Sciences, University of California, San Francisco, San Francisco, CA; 8 Departments of Acute and Chronic Care, Epidemiology and Biostatistics, George Washington University School of Nursing and Milken Institute School of Public Health, Washington, DC; 9 Division of Geriatrics, Department of Medicine, University of Wisconsin School of Medicine and Public Health and the William S. Middleton Memorial VA, Geriatric Research, Education and Clinical Center, Madison, WI; 10 Atherosclerosis Research Unit, University of Southern California, Los Angeles, CA; 11 Department of Physiology and Biomedical Engineering, Division of Nephrology and Hypertension, Division of Hematology Research, Mayo Clinic, Rochester, MN; 12 Department of Radiology, Mayo Clinic, Rochester, MN; 13 Department of Obstetrics and Gynecology, Columbia University, New York, NY; 14 Department of Medicine, Brigham and Women's Hospital, Harvard Medical School, Boston, MA; 15 Department of Obstetrics, Gynecology and Reproductive Sciences, Yale School of Medicine, New Haven, CT; 16 Department of Obstetrics and Gynecology, University of Colorado School of Medicine, Aurora, CO; 17 Phoenix Veterans Administration Health Care System, Phoenix, AZ.

**Keywords:** Cardiovascular disease, Cognition, Hormone therapy, Menopausal symptoms, Menopause, Osteoporosis

## Abstract

**Objective:**

The Kronos Early Estrogen Prevention Study (KEEPS) was designed to address gaps in understanding the effects of timely menopausal hormone treatments (HT) on cardiovascular health and other effects of menopause after the premature termination of the Women's Health Initiative.

**Method:**

The KEEPS was a randomized, double-blinded, placebo-controlled trial to test the hypothesis that initiation of HT (oral conjugated equine estrogens [o-CEE] or transdermal 17β-estradiol [t-E_2_]) in healthy, recently postmenopausal women (*n* = 727) would slow the progression of atherosclerosis as measured by changes in carotid artery intima-media thickness (CIMT).

**Results:**

After 4 years, neither HT affected the rate of increase in CIMT. There was a trend for reduced accumulation of coronary artery calcium with o-CEE. There were no severe adverse effects, including venous thrombosis. Several ancillary studies demonstrated a positive effect on mood with o-CEE, and reduced hot flashes, improved sleep, and maintenance of bone mineral density with both treatments. Sexual function improved with t-E_2_. There were no significant effects of either treatment on cognition, breast pain, or skin wrinkling. Variants of genes associated with estrogen metabolism influenced the age of menopause and variability in effects of the HT on CIMT. Platelet activation associated with the development of white matter hyperintensities in the brain.

**Conclusions:**

KEEPS and its ancillary studies have supported the value and safety of the use of HT in recently postmenopausal women and provide a perspective for future research to optimize HT and health of postmenopausal women. The KEEPS continuation study continues to pursue these issues.

The unexpected early termination of the estrogen plus progestin (E+P) portion of the Women's Health Initiative's (WHI) trial in 2002 for net harm, and of the WHI estrogen-only (ET) trial in 2004 for futility left scientists, physicians, and their patients with many concerns about the benefits and risks of menopausal hormone treatments (HT). Despite the large scale and high cost of the WHI, many questions remained unanswered and some new questions arose, the most critical being whether the WHI findings were affected by the fact that most women in the hormone trials were 5 or more years beyond their menopause on entry into the study and many had existing cardiovascular disease.^1^ As it seemed unlikely that the National Institutes of Health would fund another study to address these questions, the Kronos Longevity Research Institute, a privately funded non-profit institution located in Phoenix, AZ, brought together basic and clinical scientists, some of whom had been investigators in the WHI, from nine academic centers and one collaborating site across the country to design a new study named the Kronos Early Estrogen Prevention Study (KEEPS). The primary goal of the KEEPS was to determine if, in women without preexisting cardiovascular disease, HT begun within 3 years of a woman's last menstrual period (2 y after the World Health Organization definition of menopause) would slow the progression of subclinical atherosclerosis as indicated by changes in carotid artery intima-media thickness (CIMT). Secondary goals included evaluation of risk factors for cardiovascular disease and measurement of coronary artery calcium (CAC). Other goals included aspects of postmenopausal health, risks, and evidence for adverse side effects. This review summarizes what has been learned from the KEEPS during the past decade, including multiple ancillary studies that have provided new insights into cognitive function, mood/affective outcomes, brain structure, menopausal symptoms including sleep quality and sexual function, bone health, metabolism, biomarkers for disease processes, breast pain, and pharmacogenomics of HT. The strengths and shortcomings of these data will be discussed as they may inform current use of HT and guide future research in this field.

## STUDY DESIGN AND POPULATION

### Study design

KEEPS (NCT00154180) was a multi-center, 4-year randomized, double-blind, placebo-controlled national trial.^2^ The age of KEEPS participants (42-58 y within 6 mo to 3 y of natural menopause) was representative of the majority of women who would likely seek HT for menopausal symptoms, as were women included in many of the previous observational studies and the women within the age group of 50 to 54 years who participated in the WHI.^3,4^ This age range for KEEPS participants intentionally matched the clinically relevant portion of the WHI, excluding older women who were 55 to 80 years of age which brought the average age of WHI participants to 63.3 years. To exclude women with subclinical cardiovascular disease in KEEPS, a CAC score had to be <50 Agatston Units (AU) at the screening visit. Other exclusion criteria for KEEPS that differentiate this cohort from those of other studies included body mass index (BMI) >35 kg/m^2^, untreated hypertension, dyslipidemia (including use of statins), diabetes, history of cardiovascular disease, smoking more than 10 cigarettes a day, and a history of cancer or other major chronic diseases.^5^

The choice of HT used in KEEPS was dictated, in part, by the prescription guidelines and recommendations developed by professional societies after the WHI, specifically, to “use the lowest dose.”^6^ Therefore, the dose of oral conjugated equine estrogens (o-CEE) was selected at 0.45 mg/d rather than the higher 0.625 mg/d used in the WHI. A second treatment group received transdermal 17β-estradiol (t-E_2_; 50 μg/d), making KEEPS the first randomized, placebo-controlled trial to include two different formulations of HT in the same study of cardiovascular disease.^2^ Both treatment groups received micronized progesterone (200 mg/d) for the first 12 days each month instead of the synthetic medroxyprogesterone acetate (MPA) that was used as a continuous regime in women with a uterus in the WHI (due to concerns over the finding of increased breast cancer with the latter). Women in the placebo group were administered matching placebo pill, patch, and 12 days of a placebo capsule. It was understood that the use in KEEPS of these formulations and the dose of o-CEE and of natural progesterone, and the cyclical, instead of continuous use of the latter, differed from that in WHI and other contemporary trials, making direct comparisons of outcomes from KEEPS with those of other trials limited.

### Study cohort

A full CONSORT diagram for recruitment and inclusion is published elsewhere.^7^ In brief, of the 4,533 phone enquiries for the study, 3,455 were disqualified including those who did not complete the screening process. Of the 1,078 who consented for the study, 290 were excluded based on screening medical history or laboratory tests. The majority of KEEPS participants were non-Hispanic white (80%) and held a bachelor's degree or higher (74%). Based on clinical criteria, the KEEPS participants were free of subclinical cardiovascular disease at the beginning of the study (Table 1).

**TABLE 1:** Cardiovascular risk factors for KEEPS participant at baseline and after 48 months after randomization to placebo, o-CEE or t-E_2_^*a*^

	Baseline			48 mo		
Parameter	Placebo (*n* = 275)	o-CEE (*n* = 230)	t-E_2_ (*n* = 222)	Placebo (*n* = 221)	o-CEE (*n* = 188)	t-E_2_ (*n* = 180)
Age	52.5 (2.5)	52.8 (2.6)	52.7 (2.6)	56.8 (2.4)	57.0 (2.6)	57.0 (2.6)
BMI, kg/m^2^	26.4 (4.3)	26.0 (4.3)	26.0 (4.4)	26.8 (4.4)	26.3 (4.7)	26.1 (4.9)
Waist circumference, cm	84.8 (12.0)	84.2 (11.3)	84.0 (11.8)	86.0 (11.6)	84.3 (11.0)	83.3 (11.8)
Systolic blood pressure, mm Hg	119.8 (14.4)	119.0 (14.8)	117.4 (15.6)	118.1 (13.6)	118.9 (13.2)	116.8 (15.3)
Diastolic blood pressure, mm Hg	75.4 (9.5)	75.3 (8.3)	74.1 (9.7)	73.5 (8.7)	74.9 (8.9)	73.3 (9.8)
Total cholesterol						
mmol/L	5.4 (0.9)	5.4 (0.8)	5.4 (0.9)	5.5 (1.0)	5.5 (0.8)	5.4 (0.9)
mg/dL	207.4 (33.9)	207.7 (31.6)	209.3 (35.6)	212.0 (37.2)	210.7 (32.4)	208.0 (35.5)
LDL cholesterol						
mmol/L	2.9 (0.7)	2.9 (0.7)	2.9 (0.8)	3.0 (0.8)	2.8 (0.8)	2.9 (0.8)
mg/dL	110.9 (26.6)	110.8 (27.8)	111.0 (29.2)	114.4 (30.3)	108.4 (29.6)	111.5 (31.7)
HDL cholesterol						
mmol/L	1.8 (0.4)	1.9 (0.4)	1.9 (0.4)	1.8 (0.4)	2.0 (0.4)	1.9 (0.4)
mg/dL	70.3 (13.7)	72.9 (14.5)	73.2 (15.6)	70.8 (14.8)	76.5 (15.7)	72.8 (15.7)
Triglyceride						
mmol/L	1.0 (0.7)	0.9 (0.6)	1.0 (0.6)	1.0 (0.6)	1.1 (0.6)	0.9 (0.5)
mg/dL	91.6 (60.3)	83.8 (55.9)	84.6 (49.7)	90.7 (56.2)	98.3 (54.8)	83.9 (44.1)
Non-HDL cholesterol						
mmol/L	3.5 (0.8)	3.5 (0.7)	3.5 (0.8)	3.7 (0.8)	3.5 (0.7)	3.5 (0.8)
mg/dL	137.0 (29.7)	134.7 (28.6)	136.1 (32.6)	141.1 (32.7)	134.3 (28.3)	135.2 (31.7)
Fasting insulin, pmol/L	42.6 (43.7)	35.7 (30.5)	48.5 (91.9)	42.7 (52.9)	32.4 (35.8)	34.3 (39.1)
Fasting glucose						
mmol/L	4.4 (0.5)	4.4 (0.5)	4.4 (0.6)	4.5 (0.6)	4.5 (0.5)	4.4 (0.4)
mg/dL	79.7 (9.8)	79.2 (8.7)	80.0 (11.4)	82.0 (10.1)	81.1 (9.7)	80.2 (7.8)
HOMA-IR, unit	1.24 (1.35)	1.02 (0.94)	1.55 (3.77)	1.28 (1.54)	0.94 (1.04)	1.00 (1.18)
hs-CRP, nmol/L	22.2 (36.3)	17.2 (23.7)	21.5 (34.2)	27.6 (39.6)	31.6 (33.5)	26.8 (48.7)
IL-6, pg/mL	3.8 (9.5)	4.3 (14.6)	3.8 (13.7)	2.9 (6.1)	3.6 (12.1)	2.4 (2.7)

BMI, body mass index; HDL, high-density lipoprotein; HOMA-IR, homeostasis model assessment of insulin resistance; hs-CRP, high-sensitivity C-reactive protein; IL-6, Interleukin 6; KEEPS, Kronos Early Estrogen Prevention Study; LDL, low-density lipoprotein; o-CEE, oral conjugated equine estrogens; t-E_2_, transdermal 17β-estradiol.

^*a*^Values expressed as mean (SD).

## PRIMARY AND SECONDARY CARDIOVASCULAR OUTCOMES

### Primary outcome—changes in CIMT

CIMT was similar between women meeting inclusion criteria at screening compared with those who did not (0.726 ± 0.90 mm [*n* = 718] vs 0.725 ± 0.084 mm [*n* = 113], respectively).^5^ At baseline, CIMT correlated with age but not with menopausal symptoms or serum levels of E_2_.^8^ Of the 727 women enrolled in KEEPS, 89.3% had at least one follow-up CIMT during the treatment phase of the trial and 79.8% had a CIMT measurement at the conclusion (48 mo) of the study. The average annual increases in CIMT (0.007 mm/y) were similar across all three treatment groups (Table 2). KEEPS estimated an effect size of −0.0034 mm/y, with a group size of 145 to provide a 99% probability of detecting a difference significant at the 0.05 level.

**TABLE 2:** Carotid artery intima-media thickness (CIMT) before (baseline) and at exit from KEEPS (48 mo)^*a*^

	Baseline		48 mo		
Treatment	*n*	Mean CIMT (95% CI), mm	*n*	Mean CIMT (95% CI), mm	Estimated change^*b*^ in CIMT (95% CI), mm/y
Placebo	275	0.7213 (0.7106-0.7319)	217	0.7502 (0.7388-0.7619)	0.0072 (0.0058-0.0086)
o-CEE	230	0.7268 (0.7152-0.7384)	185	0.7591 (0.7465-0.7717)	0.008 (0.0065-0.0095)
t-E_2_	222	0.7176 (0.7058-0.7294)	178	0.7488 (0.7359-0.7616)	0.0077 (0.0061-0.0092)

o-CEE, oral conjugated equine estrogens; t-E_2_, transdermal 17-β estradiol.

^*a*^Derived from Table 2 of reference 7.

^*b*^Change between baseline and 48 months as annual slope.

### Secondary outcome—CAC

Of otherwise eligible women 14% were excluded from KEEPS due to a CAC scores >50 AU. Women excluded based on CAC score had similar CIMT versus women with CAC scores <50 AU, indicating a discordance in the type and degree of atherosclerosis. This observation of apparent discordance in the degree of atherosclerosis defined by CAC and CIMT suggests that a different cellular process and risk factors may contribute to the progression of atherosclerosis at various anatomical locations.^5^

Reflecting the CAC exclusion criterion, 87% of the women enrolled in KEEPS did not have any detectable levels of calcium in their coronary arteries before randomization (CAC scores of 0 AU at baseline). About 6% of participants had CAC scores between 1 and 5 AU and 6% had scores >5 to 50 AU (Table 3). Among all KEEPS participants who had CAC scores assessed both at baseline and after 48 months of treatment, there were no statistically significant differences in changes in CAC scores among the three treatment groups with an average increase of about 19% over the 4-year course of the KEEPS trial. The percentage of women who had CAC scores >5 at 48 months, however, trended to be less in the o-CEE group than in the placebo (Table 3). Although this trend of less accumulation of calcium in the coronary arteries in the HT groups did not reach statistical significance, it is consistent with the finding of lower CAC scores after o-CEE (albeit at a somewhat higher dose than in KEEPS) in the E-only group of the WHI^9^ and prospective assessments of CAC in postmenopausal women.^10^ As KEEPS had excluded women with more significant CAC at screening, the study was underpowered to detect statistical significance for these small changes in CAC among groups. Whether HT doses used in KEEPS or higher doses used in WHI or ELITE would have slowed CAC if KEEPS would have continued for more than 4 years remains a matter of speculation.

**TABLE 3:** The secondary cardiovascular outcome of KEEPS (coronary artery calcification, CAC) by treatment group

Baseline		48 mo	
Treatment	> 5 AU	Treatment	> 5 AU
Placebo (*n* = 275)	15.97 (5.16-55.50)^*a*^, *n* = 15 (5.5%)^*b*^	Placebo (*n* = 221)	22.69 (5.3-154.01)^*a*^, *n* = 32 (14.5%)^*b*^
o-CEE (*n* = 230)	12.72 (5.45-42.30)^*a*^, *n* = 15 (6.5%)^*b*^	o-CEE (*n* = 188)	20.66 (5.47-112.16)^*a*^, *n* = 14 (7.4%)^*b*^
t-E_2_ (*n* = 222)	13.68 (6.06-32.37)^*a*^, *n* = 15 (6.8%)^*b*^	t-E_2_ (*n* = 180)	21.65 (6.36-76.58)^*a*^, *n* = 21 (11.7%)^*b*^

^*a*^Median with 25th and 75th percentile of calcium scores in Agatston Units (AU).

^*b*^(%) of number of participants in the designated group at the designated time point.

## OTHER CARDIOVASCULAR OUTCOMES

Blood pressure did not change significantly across any of the treatment groups during the study (Table 1).

## ANCILLARY CARDIOVASCULAR STUDIES

Additional studies were conducted that provide an integrated picture of the cardiovascular health of the KEEPS cohort, and insight into potential mechanisms by which HT might affect progression of cardiovascular disease (Table 4). Despite the limited number of participants, their results constitute technical details and preliminary data from which other hypotheses can be developed and tested.

**TABLE 4:** Summary of key findings from KEEPS and KEEPS ancillary studies

Outcome	Reference
*Cardiovascular outcomes*	
• Primary outcome: neutral effect of HT on rate of change in CIMT	7
• Secondary outcome: nonsignificant trend toward reduction in CAC with both o-CEE and t-E_2_	7
• Neutral effect of HT on systolic blood pressure	7
• Negative association of brachial artery reactive hyperemia index with CIMT independent of treatment group	16
• Differential effects of t-E_2_ and o-CEE on soluble vasoactive factors released from platelets	25
• Changes in CIMT associated with the average number of MV derived from activated platelet, leukocytes and endothelium	26, 27
*Cognition and brain structure*	
• A lower cardiovascular risk profile associated with better global cognitive scores	39
• Systolic blood pressure showed a negative association with auditory and working memory	37
• No adverse effects on cognition with either o-CEE or t-E_2_	38
• Decreased depression and anxiety with o-CEE	38
• Greater expansion of ventricular volume with o-CEE than placebo	42
• Activated platelets and thrombogenic microvesicles associated with development of white matter hyperintensities	43
*Menopausal symptoms and sexual function*	
• Decreases in hot flashes, night sweats with o-CEE and t-E_2_	49
• Improvement in some domains of sleep with o-CEE and t-E_2_	53
• Improvements in physical related sexual function were greater with t-E_2_ than o-CEE	60
• Improvements in emotionally related sexual function (desire, arousal, orgasm and sexual satisfaction) were greater with t-E_2_ than o-CEE	60
*Skin health*	
• Neither total wrinkle score nor total skin rigidity score differed among treatment groups	67, 68
*Bone health*	
• Bone mineral density maintained at the spine and wrist with o-CEE and t-E_2_	69
*Breast pain*	
• No increases in breast pain with either o-CEE or t-E_2_	72
*Metabolism*	
• Trend toward improved metabolic profiles for lipids with o-CEE and for insulin resistance with t-E_2_	7
• Epicardial and pericardial fat increased with increasing BMI and components of metabolic syndrome	80
• Pericardial fat associated with increases in CAC in the t-E_2_ group	81
*Pharmacogenomics*	
• Variants of genes related to innate immunity influenced the effect of treatment on development of CIMT and CAC	82, 88
• Variants in genes for enzymes related to estrogen metabolism associated with age of onset of menopause and efficacy of HT to relieve menopausal symptoms.	87, 88

CAC, coronary artery calcification; CIMT, carotid artery intima-media thickness; HT, hormone treatments; MV, microvesicles; o-CEE, oral conjugated equine estrogen; t-E_2_, transdermal 17β-estradiol.

### Endothelial function

One of the earliest changes in vascular function associated with the development of atherosclerosis is loss of endothelium-derived nitric oxide. Nitric oxide causes vasodilatation, reduces platelet activation, and reduces adhesion of leukocytes and platelets to the uninjured vascular wall.^11^ The endothelial production of nitric oxide can be modulated by estrogen.^12^ To investigate the potential impact of HT on endothelial function in KEEPS participants at one clinical site, a noninvasive reactive hyperemia test (RHI) utilizing digital peripheral tonometry (EndoPat, model 200, Itamar Medical, Ltd., Caesarea, Israel) was used to measures changes in digital pulse volume after occlusion of the brachial artery. These changes in volume are a measure of shear stress-induced vasodilatation caused by the release of nitric oxide.^13^ In a subset of KEEPS participants (*n* = 102), the reactive hyperemic index (RHI) before randomization (baseline) ranged from 1.22 to 5.44. An RHI <1.35 has a sensitivity of 80% and a specificity of 85% to identify persons with coronary endothelial dysfunction defined by the degree of vasodilatation invoked by an intracoronary arterial infusion of acetylcholine.^14^ At baseline, before randomization to treatment, RHI did not associate with CIMT or CAC scores, suggesting that RHI measures elements of atherosclerosis independent of arterial wall thickness and calcification. As might be expected, however, the conventional cardiovascular risk factors of elevated BMI (*ρ* = −0.21, *P* = 0.031) and waist circumference (*ρ* = −0.19, *P* = 0.05) both associated inversely with RHI, an association that was absent in participants who had never smoked (*n* = 73; *P* = 0.375).^15^

Among the above participants, 83 women also had RHI measurements at their exit visit at 48 months (during the nonprogesterone phase of treatment) at which time there again was a wide variation in RHI values among women (range 1.0-4.26), which did not change significantly from the baseline in any of the three treatment groups. Among all groups combined, there was, however, a significant inverse association between the change in RHI and the change in CIMT (*P* = 0.012) but not CAC, suggesting that a decrease in endothelial function is associated with an increase in progression of subclinical atherosclerosis in carotid but not coronary arteries.^16^

These results are consistent with the general hypothesis that decreased endothelial vasodilatory function coincides with progression of atherosclerosis; however, the absence of a beneficial effect of long-term HT on reactive hyperemia in KEEPS is inconsistent with observations from other studies.^17,18^ The small sample size, choice of digital tonometry rather than a more sensitive measure of reactive hyperemia by ultrasound of the brachial artery, as well as the low doses of HT utilized in KEEPS may account, in part, for the discrepancy.^19^

### Blood elements and cell-derived microvesicles

Activation of platelets and migration of monocytes/macrophages into the vascular wall at regions of endothelial dysfunction or damage are initiating steps in the development of atherosclerotic lesions.^20-22^ In a subset of KEEPS participants at their baseline visit, in vitro measures of platelet aggregation and granular secretion showed statistical association with individual components of the metabolic syndrome (waist circumference, systolic blood pressure, fasting glucose, high-density lipoprotein cholesterol and triglycerides). At the baseline visit, the number of platelets in the blood was positively associated with increasing waist circumference, but not with other components of the metabolic syndrome, whereas platelet secretion of adenosine triphosphate and expression of P-selectin were inversely associated with increasing blood glucose and blood pressure.^23^ Thus, a lack of associations of the “metabolic syndrome” as an index or summary measure based on recommended cutoff scores of the five elements may mask associations of its individual components. Identifying the individual components of the metabolic syndrome associated with platelet activation may help to target preventive strategies on manageable or treatable cardiovascular risk factors.

Circulating platelets do not contain a nucleus, but their megakaryocyte precursors do and, thus, represent the target for genomic effects of the sex steroids on the characteristics of the circulating platelet pool.^24^ Although there were no statistically significant differences in number, aggregatory or secretory capacity of platelets among KEEPS groups over the course of the treatment period,^25,26^ the content of vasoactive and mitogenic factors derived from the platelet lysate decreased within the HT groups compared with the placebo group.^25^ In addition, the platelet content of 5-hydroxytryptamine and vasoactive prostanoids (prostacyclin and thromboxane) differed between the o-CEE and t-E_2_ groups possibly contributing, in part, to higher risk of thrombotic events with oral than with transdermal HT.^27,28^ Collectively, differences in the amount and types of vasoactive and mitogenic factors derived from the activated platelets could influence vascular remodeling, such as increases in CIMT, within the vicinity of platelet activation.

Activated platelets, leukocytes, and endothelial cells release membrane-bound vesicles less than 1 μm in size called microvesicles (MV). These MV carry bioactive molecules (DNAs, RNAs, proteins, and metabolites) capable of affecting distance cells.^29^ At the baseline KEEPS visit, numbers of platelet-derived and procoagulant MV were directly associated with CIMT but not CAC or RHI,^23^ whereas monocyte- and endothelial cell-derived MV were associated with systolic blood pressure, which was also associated with CIMT. These observations open new insight into how activation of cells within the vascular compartment may contribute to the progression of vascular lesions.^26,30^

In general, the number of circulating MV varies inversely with serum levels of 17β-E_2_ after menopause.^31^ After 48 months of treatment, the number of circulating MV did not, however, differ across HT groups in KEEPS, which might reflect the rather narrow range in serum E_2_ levels among groups despite the different treatments.^7^ The average change in CIMT correlated with the average number of MV derived from leukocytes and vascular endothelium, and those expressing cell adhesion molecules, an observation consistent with that before treatment and with the general hypothesis that intravascular cellular activation occurs during vascular remodeling of the carotid arteries. These findings also imply selective influences regulating the number of both types of MV, rather than a simple numerical relationship. Although development of MV as biomarkers for active disease processes may not herald increases in CIMT, the diagnostic potential of specific populations of MV to detect elevation in CAC, particularly those MV with thrombin generating capacity, may be most useful. Effects of HT on MV derived from stem cells and adipocytes should be explored in more detail in larger cohorts.^32,33^

## ADVERSE CARDIOVASCULAR EVENTS

There were no venous thrombotic events and the single myocardial infarction was reported in a woman assigned to t-E_2_, but it occurred before the start of treatment.^7^

## GENERAL EFFECTS OF HT ON POSTMENOPAUSAL WOMEN IN KEEPS

### Cognitive function and mood

Before the publication of results from the WHI ancillary Memory Study (WHIMS), there was conflicting but generally favorable evidence regarding effects of HT on cognitive function and risk of Alzheimer's disease.^34^ The WIHMS was a substudy restricted to women in the WHI who initiated therapy at greater than 65 years of age. The initial report from WHIMS found that HT increased the risk for dementia and was most pronounced for therapies using MPA in addition to o-CEE.^35^ The end conclusions were based on a small number of participants and raised many concerns regarding the experimental design.^36^ These observations influenced the design of the KEEPS cognitive and affective substudy (KEEPS Cog),^37^ which was based on the underlying hypothesis of a “critical period” in which initiation of HT in the early postmenopausal phase might benefit or protect brain health. Furthermore, questions remained after WHIMS regarding the influence of the type of estrogen and the use of synthetic progestogens on cognition. Therefore, the basic design of KEEPS that enrolled women within 3 years of menopause, with the inclusion of both o-CEE and t-E_2_, with the use of natural and cyclic progesterone, provided an ideal background to examine HT effects on cognition. KEEPS Cog was funded by National Institutes of Health and was open to all KEEPS participants. A set of 19 cognitive measures (many of which were included in WHIMS) were selected to examine intelligence, verbal learning and memory, language and mental flexibility, attention and executive function, working memory, and mood. The tests were administered at four time points: baseline and 18, 36, and 48 months after randomization. The 36-month visit was to be conducted within days 6 to 12 during the administration of progesterone.^37^

Of the 727 initial enrollees in KEEPS, 693 enrolled in the KEEPS Cog study^38^; 220 were randomized to o-CEE, 211 were randomized to t-E_2_, and 262 were randomized to placebo. The demographics of women participating in the KEEPS Cog were the same as those for the parent KEEPS study.^38^ Baseline cognitive data collected before randomization were assessed relative to latent class membership defined by cardiovascular risk profiles (blood pressure, blood lipids, insulin resistance, and Framingham risk score) and relevant sample characteristics (education, apolipoprotein E ε4 [APOEε4] status, ethnicity and age). The latent profile analysis identified two distinct phenotypical classes of cardiovascular risk: low risk, representing 62% of enrollees and high risk, representing 38% of enrollees. Baseline performance of low-risk women on language and mental flexibility tasks and global cognition was better than that of high-risk women. Education level was associated inversely with risk classification, and older age and Hispanic ethnicity increased the probability of being in the high-risk group. The presence of the *APOE*ε4 also was more prevalent in the high-risk group.^39^

Associations for specific cardiovascular risk factors that might affect cognitive performance were investigated in 571 women for whom a complete data set and *APOE*ε4 genotypes were available. After controlling for age, education and *APOE*ε4 status, systolic blood pressure showed an inverse association with performance on auditory attention and working memory, a relationship that was not related to endogenous hormone levels at baseline.^40^ These results are consistent with the growing body of evidence for a relationship between increased systolic blood pressure and with both adverse structural changes in the brain and decreased cognition.^41^

A total of 662 women provided sufficient neuropsychological test data to be included in the longitudinal analytic sample.^38^ Effects of HT on changes in cognition and mood were analyzed using linear mixed-effects models. Longitudinal outcome measures included the following: Modified Mini-Mental State examination; four cognitive latent factors (verbal learning/memory, auditory attention/working memory, visual attention/executive function, and speeded language/mental flexibility); and mood measured by the Profiles of Mood States (POMS). There were no treatment-related effects found for any of the cognitive outcomes. Regarding mood, however, women treated with o-CEE but not t-E_2_ reported less depression and anxiety symptoms compared with placebo.^38^ It should be emphasized that these results are limited to the 4 years of HT in women with low baseline cardiovascular risk. Long-term follow-up of women enrolled in KEEPS is currently underway to evaluate these effects in women after cessation of HT. Follow-up of younger WHI participants (aged 50-55 y at HT onset) was conducted in the E-only WHI trial. Using methods similar to the WHIMS substudy, no sustained benefit or risk was found on cognitive function, although these women were examined 7 years after cessation of HT, and no information was obtained regarding continuation or early discontinuation of treatment.^35^

### Brain structure

An ancillary brain magnetic resonance imaging (MRI) study was conducted on the KEEPS Mayo Clinic cohort (*n* = 118) to investigate the effects of o-CEE and t-E_2_ on changes in brain structure over the 4 years of KEEPS, among whom 5 participants were excluded due to neurological disorders or MRI contraindications and 12 women declined to participate. Of the remaining 101 women who underwent an MRI scan before treatment, MRI data were analyzed from 95 participants with at least one follow-up MRI examination during the course of the study at month 18 (*n* = 92), 36 (*n* = 87), or 48 (*n* = 79). The major finding of this study was that the rates of ventricular volume increases were greater in women who received o-CEE compared with those receiving placebo. Although the rates of changes in these structural MRI measures did not differ between the o-CEE and t-E_2_ groups, the rates of increase in ventricular volume did not reach statistical significance in the t-E_2_ group, compared with placebo, perhaps due to our limited sample size and short follow-up. Increases in ventricular volume over 18 months of treatment were greater in the oral CEE group if HT was initiated later in menopause (although the time differences were small given the narrow postmenopause recruitment window of KEEPS). Changes in structural MRI measures were not accompanied by significant differences in global cognition within or between groups.^42^ The volume of white matter hyperintense (WMH) lesions increased during the treatment phase of the trial and was not associated with treatment assignment. The WMH lesion volume, however, correlated with the platelet-derived, thrombogenic microvesicles at baseline, suggesting that in vivo platelet activation may contribute to a cascade of events leading to the development of WMH lesions in recently postmenopausal women.^43^

### Menopausal symptoms

Menopausal symptoms are linked to overall health and disease risks, as well as to impaired quality of life (QOL), in most^44-47^ but not all^48^ studies. Women in KEEPS (*N* = 727) completed brief symptom questionnaires at 6, 12, 24, 36, and 48 months.^49^ KEEPS, self-reported symptoms of hot flashes, vaginal dryness/dyspareunia, mood swings, sleep, and palpitations were monitored, and linked to baseline health status and improvements over time. The use of both o-CEE and t-E_2_ also permitted direct comparisons between these two widely used forms of hormone therapy.

At baseline, menopausal symptoms were not associated with markers of subclinical cardiovascular disease (CIMT and CAC).^8^ Depressive symptoms and self-reported palpitations tended to associate positively with CAC, and although this association did not achieve statistical significance, it may be worthy of further study in larger cohorts. Symptoms of hot flashes/night sweats, irritability, and insomnia at baseline were more likely among black women, with a very large odds ratio for irritability (19.23, 95% CI, 11.72-31.57), a striking finding that requires confirmation and further exploration in larger cohorts.

Moderate-to-severe symptoms of hot flashes and night sweats were similarly reduced by both o-CEE and t-E_2_ compared with placebo.^49^ There was no differentiation of either HT versus placebo on relief of symptoms of irritability or mood swings. There was a modest, nonsignificant overall trend toward symptom improvement over time in the placebo group, not associated with BMI or race/ethnicity.^49^ These latter findings confirm a natural time course for regression of common menopausal symptoms of hot flashes, night sweats, irritability, and mood swings over time for most but not all women.^50,51^

### Circulating estrogen levels

In addition to examining the role of HT in addressing symptoms, KEEPS measured E_2_ (the principal circulating hormone resulting from t-E_2_) and estrone (E_1_) in a subset of participants (*N* = 426). Using a well-validated, immune-radiometric assay,^7^ which is highly sensitive and correlates with the “gold-standard” mass spectrometry method.^52^ At baseline, there was no relationship between circulating E_2_ levels and symptoms, but among women reporting complete relief of hot flashes, those randomized to t-E_2_ had a mean E_2_ level of 44 pg/mL (95% CI, 39-50) compared with those reporting moderate or severe persistence of hot flashes, who had circulating E_2_ levels of 9 to 11 pg/mL (95% CI, 5-23). This difference is statistically significant and clinically relevant, in that it helps define a clinically effective window of E_2_ levels when using t-E_2_.

### Sleep quality

Sleep quality was examined both at baseline and in response to treatment assignment.^53^ The Pittsburgh Sleep Quality Index (PSQI) was used for assessments at baseline, and 6, 18, 36, and 48 months.^54^ A global PSQI score of >8, consistent with disturbed sleep, was reported by 24% of women at baseline. Sleep quality improved with both o-CEE and t-E_2_. Individual sleep domain scores were improved similarly by o-CEE and t-E_2_ for sleep satisfaction and latency; however, the sleep disturbances domain was improved by t-E_2_, but not o-CEE, suggesting a possible superiority of t-E_2_ for this aspect of sleep. Taken together, these findings provide new and useful information regarding the effects of HT on menopausal symptoms and quality of life. These data suggest a therapeutic equivalence of 0.45 mg of CEE to 50 μg of t- E_2_—although with some distinction between the two.^51^

### Sexual function

Estrogen deficiency impacts both physical and psychological domains that are critical to sexual experience. Estrogen insufficiency is associated with anatomical changes that contribute to sexual dysfunction, including thinning of the skin of external genitalia, loss of subcutaneous fat, parallel involution of the corpora cavernosa, as well as thinning of the epithelium lining the vagina.^55-58^ Together these changes can lead to reduced lubrication and pain or discomfort during intercourse. As estrogen is a modulator of serotonergic function, it affects regions of the brain known to regulate mood and desire, including the amygdala, hippocampus, and the hypothalamus.^59^ Estrogen loss is associated with alterations in mood, sleep, and cognitive function, all directly or indirectly influencing sexual function.^55^

*Effects of o-CEE versus t-E*_*2*_: Improvements in domains of sexual function have been previously noted with the use of HT; however, the effects of o-CEE versus t-E_2_ have not previously been directly compared. Five hundred and seventy-five of the 727 KEEPS enrollees agreed to participate in the ancillary study of sexual function.^60^ Participants completed the Female Sexual Function Inventory (FSFI), a well-validated 19-item questionnaire for assessing the key dimensions of sexual function along six domains of sexual function, including desire, arousal, lubrication, orgasm, satisfaction, and pain.^61,62^ Treatment with t-E_2_, but not o-CEE, was associated with a significant improvement in the FSFI overall score across all time points compared with placebo. In the individual domains of sexual function, t-E_2_ and o-CEE both differed from placebo. Symptoms related directly to tissue effects of estrogens on the reproductive track, such as lubrication and pain on penetration, demonstrated a progressive exacerbation with time, yet were reduced with the use of either t-E_2_ or o-CEE. In contrast, the more subjective domains of desire, arousal, orgasm, and sexual satisfaction demonstrated a relatively steady state over time, yet the superiority of t-E_2_ over o-CEE was apparent in the greater efficacy of t-E_2_ in improving these nonphysical sexual function aspects.

*Steroid hormone binding*: Levels of sex hormone-binding globulin were higher in the o-CEE compared with the t-E_2_ and placebo groups.^7^ Given that hepatic sex hormone-binding globulin controls levels of free testosterone,^63^ it may not be surprising that these differing formulations of HT might influence sexual function differently. Free testosterone may be critically important to desire, arousal, orgasm, and sexual satisfaction. Overall, the proportion of women with low sexual function was significantly decreased after treatment with t-E_2_, but not o-CEE compared with placebo. Thus, KEEPS findings suggest that postmenopausal women with sexual concerns are better served by treatment with t-E_2_ than with o-CEE.

### Skin health

Skin wrinkling is another consequence of aging that is believed to be related to menopause and possibly to estrogen deprivation.^64^ Estrogens influence skin appearance through regulation of dermal cell functions such as collagen production and hydration. In retrospective trials, long-term HT users were shown to have more elastic skin and less severe wrinkling than women who never used HT.^65^ KEEPS evaluated facial wrinkling^66^ at 11 locations on the face and neck and skin rigidity (using a durometer) at the forehead and cheek in an ancillary study conducted at a subset of KEEPS sites at baseline and yearly for 4 years.^65,67^ Among the 106 women assessed at baseline, skin wrinkling was noted to be lowest among black women. Skin rigidity was not associated with race/ethnicity. Increasing weight was associated with less skin wrinkling, and, among white women, age was associated with wrinkling, and time since menopause was associated with increased skin rigidity. Waist circumference was also positively associated with skin rigidity. Wrinkling was associated with a past history of smoking, but not with current smoking. These relationships were not modified by sun exposure, but overall numbers in this study were small.^67^

Longitudinal evaluation of skin wrinkling and rigidity was completed in 116 women.^68^ Neither total wrinkle score nor total rigidity score was significantly different at the end of the 48 months among women randomized to o-CEE, t-E_2_, or placebo. Although black women had the lowest wrinkle scores compared with white women across all 4 years, skin rigidity decreased in all groups over time, but the decrease in total facial rigidity was significantly less in black women compared with white women after 4 years. Discrepancies between this randomized trial and retrospective studies could be due to selection bias inherent in retrospective studies or because the timing of skin changes are slow and may take longer than would be detectable in a 4-year trial. Differences from other studies may also relate to proportion of women who were current smokers or other lifestyle choices including sun exposure.

### Bone health

Efficacy of HT to prevent loss of bone mineral density was assessed in a subgroup of KEEPS participants (*n* = 76) by quantitative computed tomography (from the CAC scans) to assess bone geometry and volumetric bone mineral density at the thoracic and lumbar spine, by dual-energy x-ray absorptiometry at the femoral neck, and by high-resolution peripheral quantitative computed tomography of trabecular and cortical bone at the distal radius. In addition to efficacy of the lower doses of HT used in KEEPS to reduce hot flashes and improve sleep, as expected based on previous studies, both treatments maintained density of trabecular and cortical bone of the spine and hip compared with placebo.^69^ These results are consistent with the WHI, which showed that HT protects against femoral neck fracture.^70^

### Breast health

Breast pain is common in postmenopausal women. In the WHI, there was a higher reported incidence of breast pain in women randomized to 0.625 mg/d o-CEE either with or without MPA compared with those randomized to placebo.^71^ Using a modification of the McGill Pain Questionnaire, there were no increases in reported severity of cyclic or noncyclic breast pain in KEEPS participants at the Mayo Clinic randomized to o-CEE or t-E_2_ (*n* = 113).^72^ The lack of increase in breast pain in KEEPS may reflect the lower dose of o-CEE and the transdermal product compared with higher o-CEE in the WHI, but selection bias may have also contributed as KEEPS participants were required to have a normal mammogram within a year of enrollment into the study. The number of new cases of breast cancers in the main KEEPS study was small and the incidence did not differ among groups.^7^

### Metabolism

Given the rigorous inclusion and exclusion criteria, KEEPS participants had low metabolic cardiovascular risk profiles based on BMI, serum lipids, triglycerides and homeostasis model assessment of insulin resistance (HOMA-IR), and inflammatory markers of interleukin-6 and c-reactive protein, which did not differ significantly among the randomized groups (Table 1).^5,7^ As expected, in the o-CEE group, low-density lipoproteins cholesterol decreased, whereas total and high-density lipoprotein cholesterol and triglycerides increased compared with the placebo group, while results in the t-E_2_ group tended to be intermediate except for the lack of increase in triglycerides (Table 1). These changes in lipoprotein cholesterol are consistent with direct hepatic effects expected with oral HT as was reported in other studies.^73,74^ Insulin resistance tended to decrease in both hormone groups, consistent with a decrease in risk of new-onset diabetes in the WHI, but which also requires additional analysis relative to other cardiovascular risk factors (Table 1) and individual changes in atherosclerosis in KEEPS.

*Orexin-A*: An ancillary study of a subset of KEEPS participants who were compliant to treatment and for whom both baseline and 48 months plasma samples were available (*n* = 74) assessed orexin-A levels, a neuropeptide implicated in regulation of food intake and energy expenditure.^75,76^ At baseline, plasma levels of orexin-A were similar among the treatment groups but showed wide variation with the 25th and 75th percentiles ranging from 1.78 to 6.11 ng/mL. This variability persisted over the 48 months of treatment with levels in the o-CEE group increasing significantly more than the nonsignificant changes observed in either the t-E_2_ or placebo groups. BMI increased significantly more in the placebo group compared with both HT groups. There was, however, no correlation between changes in BMI and changes in orexin-A in any of the groups.

*Fat accretion and distribution*: There is conflicting evidence regarding the contribution of pericardial and epicardial fat to incidence of coronary artery disease and CAC.^77-79^ Amounts of epicardial, pericardial, and hepatic fat were measured from women being screened for KEEPS who had sufficient images of the liver and spleen from the computed tomographic scans performed to measure CAC to calculate hepatic fat (*n* = 652), and these were compared with BMI. Blood levels of adipokines (leptin, soluble leptin receptor, and high molecular weight adiponectin) were measured by enzyme-linked immunosorbent assay and were compared relative to BMI ≥25 kg/m^2^ and to none, ≤1 component or ≥2 components of the metabolic syndrome (waist circumference, blood pressure, fasting glucose, low levels of high-density lipoproteins, and triglycerides).^80^ Epicardial and pericardial fat increased while hepatic fat decreased with increasing BMI and components of the metabolic syndrome. Leptin increased, whereas soluble leptin receptor and adiponectin decreased with increasing BMI and metabolic syndrome characteristics. These results support a continuum of risk with increasing BMI, waist circumference, blood pressure, fasting glucose, and triglycerides.^80^ Furthermore, they suggest that assessment of individual components rather than the collective heterogeneous grouping to define “metabolic syndrome” may provide information for more targeted preventive strategies.

A cross-sectional analysis was performed on a sub-group of KEEPS participants who had CAC measurements available before and after 48 months of randomized treatment (*n* = 474). In a preliminary analysis presented at the 2018 meeting of The North American Menopause Society,^81^ epicardial fat depots increased significantly in the placebo group but not in the hormone groups after 48 months of treatment. Preliminary analysis suggests that greater pericardial fat was associated (*P* = 0.02) with greater risk for progression of CAC but only in the t-E_2_ group. Further analysis is, however, needed to determine relationships among these fat depots, conventional cardiovascular risk factors, and CAC in postmenopausal women.

## GENETIC VARIANCE AND TREATMENT EFFECTS

The KEEPS studied whether certain genetic variants influenced the effect of HT on specific outcomes, that is, CIMT, CAC, menopausal symptoms, and so on. There are multiple genetic variants potentially affecting response to estrogen, including variants in genes regulating estrogen metabolism and receptors, as well as variants of genes containing estrogen response elements in their promoter regions that have the potential to influence the effect of treatment, specifically HT, on all of the outcomes in KEEPS. A targeted candidate genetic analysis was used to gain insight into processes contributing to the main outcomes of KEEPS. This analysis included genetic analysis of 610 participants of KEEPS and consisted of a set of 13,229 single nucleotide polymorphisms (SNPs) within 764 genes from anticoagulant, procoagulant, fibrinolytic, and innate immunity pathways, and variants of estrogen receptors α and β.^82^

### Genetic variance and CIMT

After adjusting for age and pulse pressure, variables that associated positively with CIMT at baseline, SNPs of the mitogen-activated protein kinase 4 (MAP4K4 on chromosome 2) and of the interleukin 5 (IL5 on chromosome 5) genes correlated positively with CIMT (*β* = 0.03697, *P* = 2.36E-06 and β = 0.05122, *P* = 5.02E-05, respectively). Two variants for chemokine ligand 5 (CCL5 or RANTES on chromosome 17) gene correlated negatively with CIMT (*β* = −0.0427, *P* = 3.59E-05). All of these genetic variants are related to regulation of innate immunity responses.^82^

In 606 women who completed KEEPS and were compliant to treatment (placebo = 194, t-E_2_ = 161, o-CEE = 157), none of the top SNPs within the innate immunity pathway that associated with CIMT before treatment were among those that associated with CIMT after treatment. There was a, however, significant interaction between genetic variants in genes of the innate immunity pathway and treatment on the changes in CIMT over the 4 years of the trial. Although individual SNPs did not reach statistical significance with changes in CIMT during the treatment period, collectively considering all of the SNPs evaluated and the frequency of the allele in the population, these pharmacogenomic effects could account for the increase in the overall variance observed in CIMT and reduce the ability to observe a specific effect by treatment.^83^ The contribution of genetic variants within the innate immunity pathway to progression of CIMT is consistent with the overall hypothesis of an inflammatory component of the disease demonstrated by changes in the biomarkers (activated cells, platelets, and microvesicles) measured in KEEPS and by observations from other genetic studies.^84^

### Genetic variance and CAC

After adjusting for waist circumference, interleukin-1 receptor-associated kinase-2 (IRAK2 on chromosome 3) and serpin family A member 1 (SERPINA 1 on chromosome 14) associated positively with CAC; ABO on chromosome 9 associated negatively with CAC. These genes code for enzymes involved in the breaking down of elastase and posttranslational modification of proteins including those related to thrombosis.^82^ There were no associations of SNPs by treatment effect on increases in CAC >5 AU.^83^ The inability to identify any of these candidate genes with CAC most likely reflects the paucity of positive CAC data (CAC >0 AU) as well as the complexity of processes involved with cellular differentiation and mineralization, and perhaps also the interaction of metabolic and immunological factors on the calcification process.^85-87^

### Genetic variance and menopausal symptoms

To better understand factors accounting for individual variation in response to HT, two genes associated with estrogen metabolism were evaluated in a subset of KEEPS participants. The gene *SULT1A1* encodes the enzyme sulfotransferase family 1A member 1 (SULT1A1) that sulfates estrone and 17β-E_2_. This enzyme is ubiquitous affecting the circulating ratios of these estrogens to their sulfated conjugates. *SULT1A1* is polymorphic, that is, there are multiple variations in copy number and SNPs. The number of SNPs and gene copies associate with enzyme activity, thus making it a reasonable candidate gene to study variation in responses to HT. In a subset of 155 women screened for KEEPS, 8 women had a single copy, 101 women had two copies, 37 women had three copies, and 9 women had four copies of the gene. Of the SNPs evaluated, the number of G alleles at rs9282861 associated with younger age at menopause, and women with 4 G alleles had less severe insomnia and lower frequency of night sweats,^88^ which may reflect effects on melatonin metabolism.^89^

After treatment, as described above, menopausal symptoms decreased in the HT groups. The *SULT1A1* did not associate with total number of symptoms nor did the rs9282861variant associate with estrogen levels in any of the HT groups; however, in women randomized to o-CEE, the ratio of unconjugated to conjugated estrogens was lower with increasing number of variants. These differences in effects of the genetic variants on circulating ratios of the sulfonated estrogen may reflect, in part, that the o-CEE formulations contains significant amounts of these estrogen conjugates.

### Genetic variance and hormone metabolism

In addition to sulfation of estrogens, their transport into the liver, particularly of estrone sulfate, is by the membrane-bound, sodium-independent organic anion transporter protein (OATP1B1) encoded by *SLCO1B1* gene. Variant rs4149057 of this gene has been studied in relationship to statin-induced myopathy and breast cancer. In an exploratory study of 100 KEEPS participants, 75 women were homozygous for the TT allele (normal function), 24 were heterozygous for the CT allele and one woman was homozygous for the CC allele (reduced function). Before randomization, this genetic variant was not associated with total menopausal symptoms, or disaggregated scores for hot flashes, night sweats, or insomnia.^90^ After treatment, although the variant did not associate with either total symptom score or scores for hot flashes or insomnia, the reduction in number of serve night sweats was greater in women assigned to HT with the CT genotype compared with those with the TT genotype. Women with the CT genotype had higher levels of sulfonated E_2_ and E_1_ than those with the TT genotype especially in those assigned to t-E_2_. Women assigned to t-E_2_ also reported greater alleviation of sleep disturbances which may include those triggered by night sweats.^53^

Despite the fact that these studies of genetic variants with responses to HT were obtained on small numbers of women, the women were well characterized allowing for these explorations in the absence of comorbidities. In addition, the direct comparisons of effects of the genetic variants on responses to two different but clinically relevant HT formulations provide the background upon which to develop a genetic screen or algorithm that would help to personalize choice of dosing and formulation of HT for postmenopausal women in the future.

## CONCLUSION AND THE FUTURE

The KEEPS and its numerous ancillary studies have taught us many things regarding the effects of two HT methods commonly used in contemporary clinical practice in generally healthy postmenopausal women (Table 4). Although KEEPS can be criticized, as have other studies, for not being generalizable to other groups, it is critical to remember that randomized clinical trials are specific to their study populations, and it is not permissible to generalize or to formulate treatment regimens based on one to other populations.^91^ Thus, KEEPS has provided valuable information regarding a carefully characterized group of recently menopausal women for whom HT would be beneficial to manage symptoms (hot flashes, sleep disturbances, sexual function) and diseases of menopause, specifically reduction in bone loss during treatment with no demonstrable effect on cardiovascular risk factors or negative impact on cognition.

In contrast to other reported studies, KEEPS participants had to be free of subclinical coronary atherosclerosis at trial entry (ie, CAC score <50 AU), were younger (53 y) than women enrolled in the E+P trial of the WHI (63 y), and they also tended to have lower body mass index, systolic blood pressure, total cholesterol, triglycerides, and fasting blood glucose, but higher high-density lipoprotein-cholesterol (HDL-C) levels (Table 1).^5^

KEEPS also differed from the Early versus Late Intervention Trial with Estrogen (ELITE), which purposefully included two strata of women (<6 y since menopause and >10 y since menopause when randomized). Like the WHI but unlike KEEPS, ELITE participants were not screened for subclinical atherosclerosis by CAC imaging before randomization, and may have undergone hysterectomy or bilateral oophorectomy.^92,93^ Thus, women in the early postmenopausal strata of ELITE averaged about 3 years older (average age 55 y) and 3 years from their last menstrual period compared with an average of 52 years of age and 1.5 years from the last menstrual period for women in KEEPS all of whom underwent natural menopause. In the early postmenopausal stratum of ELITE, the annual increase of CIMT (averaged 0.75 mm before randomization) was, however, only 0.0044 mm per year in the E_2_ group compared with 0.00787 mm per year in the placebo group, a rate comparable to that in the combined groups of KEEPS.^94^

This difference may be due, in part, to the type of HT used in each study. In ELITE, the HT was oral (1 mg/d) 17β-E_2_ with vaginal progesterone, whereas in KEEPS, the HT was transdermal 17β-E_2_ (50 μg/d) or low-dose oral CEE (0.45 mg/d) with oral progesterone. HT regimes in both KEEPS and ELITE were cyclic (the progesterone was not taken every day), whereas the WHI used a continuous daily synthetic progestin regime for women with a uterus. These differences between KEEPS and ELITE in chronological and menopausal age of the women, their ovarian status, formulations, doses, and mode of delivery of the HT may all be relevant to the observed differences in the primary outcomes of these clinical trials.^95^

The oral 17β-E_2_ used in ELITE had greater metabolic effects on lipid metabolism than the transdermal product used in KEEPS. The mixed estrogens and androgens in CEE are expected to have both agonist and antagonistic effects on some signaling estrogen receptor signaling pathways in light of known differences in affinity for and actions of these various elements for the estrogen receptors in the liver and on cells in the blood and vascular wall.^96,97^ Other differences in formulations of estrogen and cardiovascular outcomes from other clinical studies have been reviewed elsewhere.^98^

The conclusions from the original WHI^99^ led to many women not receiving prescriptions for estrogen even with moderate-to-severe symptoms,^100,101^ and decreased funding for further studies. Since the publication of the primary KEEPS results, other analyses of the WHI and several meta-analyses have shown that younger women, similar to KEEPS participants, demonstrate no harm from HT and may benefit in terms of cardiovascular disease risk.^94,102-108^ Clinicians should be encouraged by these results to help better characterize the benefits and risk of HT for their patients.

### More research is needed

Investigators should be encouraged to continue to obtain and evaluate data from the KEEPS cohort. KEEPS provides a defined cohort in which to study healthy aging and the subsequent cardiovascular and cognitive risks after cessation of HT.^109-113^ Indeed, the National Institutes of Aging has funded a continuation of KEEPS (NCT03718494) that will expand these preliminary studies, allowing all former KEEPS participants to enroll for follow-up assessment of cognition and brain structures, including assessments of brain volumes, white matter hyperintensities, and β-amyloid load. Furthermore, imaging of tau deposition, another marker for Alzheimer's disease-related pathology, will be performed in a subset of these women. Enrollment is ongoing and it is anticipated for the study to be completed in 2023. Results of these follow-up evaluations will provide important confirmation of physiological consequences after cessation of HT in women from KEEPS. With ongoing extended follow-up of the KEEPS cohort, it is expected that the KEEPS will “keep on giving” important data upon which to base clinical recommendations for postmenopausal women as they age. The number of aging women and the development of hormone-active agents are growing. Funding for studies of new modalities against specific aspects of menopause (anti-bone loss, anti-hot flashes, etc.) are needed so that effectiveness of those modalities can be compared with the general value of the wide systemic effects of estrogen-based HT.
